# Order Reconstruction in a Nanoconfined Nematic Liquid Crystal between Two Coaxial Cylinders

**DOI:** 10.3390/ma8125446

**Published:** 2015-11-30

**Authors:** Xuan Zhou, Zhidong Zhang, Qian Zhang, Wenjiang Ye

**Affiliations:** Department of Physics, Hebei University of Technology, Tianjin 300401, China; zhouxuan198536@163.com (X.Z.); zhangq7907@126.com (Q.Z.); wenjiang_ye@hebut.edu.cn (W.Y.)

**Keywords:** liquid crystal, order reconstruction, disclination loop, cylindrical wall, biaxial transition, Landau–de Gennes theory

## Abstract

The dynamics of a disclination loop (s = ±1/2) in nematic liquid crystals constrained between two coaxial cylinders were investigated based on two-dimensional Landau–de Gennes tensorial formalism by using a finite-difference iterative method. The effect of thickness (*d* = *R*_2_ − *R*_1_, where *R*_1_ and *R*_2_ represent the internal and external radii of the cylindrical cavity, respectively) on the director distribution of the defect was simulated using different *R*_1_ values. The results show that the order reconstruction occurs at a critical value of *d*_c_, which decreases with increasing inner ratio *R*_1_. The loop also shrinks, and the defect center deviates from the middle of the system, which is a non-planar structure. The deviation decreases with decreasing *d* or increasing *R*_1_, implying that the system tends to be a planar cell. Two models were then established to analyze the combined effect of non-planar geometry and electric field. The common action of these parameters facilitates order reconstruction, whereas their opposite action complicates the process.

## 1. Introduction

The equilibrium configuration of a confined nematic liquid crystal (NLC) is caused by interplay among surface interaction, elastic distortion, and finite-size effect. Confined systems that suffer from continuous symmetry-breaking transitions often display topological defects [1,2,3,4], which are utilized in new-generation LC devices. Therefore, study of defects is an important field in physics. In particular, defects in nanoconfined LC cells have received increased research attention. A weak local perturbation in an LC confined in a nanoscale cell can stimulate an apparent mesoscopic or even macroscopic response [5].

Eigenvalue exchange/order reconstruction occurs under severe confinement in the presence of antagonistic anchorings, and this mechanism relaxes surface-induced frustrations. Order reconstruction structures have been investigated in detail [6,7,8,9,10,11] for various boundary conditions in nematic cells bounded by parallel walls, for which the characteristic linear size of the confining plates is large and virtually infinite compared with the cell thickness. These structures are stable in a sufficiently thin cell [6,7], whose gap is comparable with the order parameter length ξ_0_ (characteristic length for order-parameter changes), or when a sufficiently strong electric or magnetic field is applied [8,9]. Studies [12,13,14] revealed that mesoscopic Landau–de Gennes theory can accurately predict the structural and phase behavior of severely confined LCs.

Although LC defects have been intensively studied theoretically and experimentally, LC shells have been rarely investigated. LC shells have received increased research interest because of their potential to generate colloids with a valence, which can be used to build colloidal architectures for photonic applications. Several scholars have numerically simulated the possible defect configurations in nematic and smectic shells [15,16,17].

NLCs in a cylindrical cavity have been studied for approximately 40 years. Cylindrical geometry is the most convenient geometry after planar cell geometry for theoretical and experimental analyses. As such, all experimentally observed and theoretically possible configurations of the director field $\vec{n}$ yield a complete or approximate analytic description [18]. The possible equilibrium configurations of an NLC in a cylindrical cavity primarily depend on how $\vec{n}$ is anchored to the cylinder surface.

In this study, we investigated the structural behavior of a nanoscale NLC system confined in a cylindrical cavity with a topological defect loop by using the finite-difference iterative method. The outline of the paper is as follows. In Section 2, we introduce the phenomenological model employed and describe the geometry of the problem and our parametrization. The results are presented in Section 3, and the conclusions are summarized in Section 4.

## 2. Theoretical Basis

### 2.1. Free Energy

Our theoretical argument is based on Landau–de Gennes theory [19], wherein the orientational order of LC is described by a second-rank symmetric and traceless tensor [3]:

(1) $$Q=\sum_{i=1}^{3}\lambda_{i}{\vec{e}}_{i}⊗{\vec{e}}_{i}$$

where ${\vec{e}}_{i}$ is the orthogonal unit vector representing the eigenvector of *Q*, and λ*_i_* is its eigenvalue. The range of eigenvalues must be $\lambda_{i}\in \left(-\frac{1}{3},\frac{2}{3}\right)$ to interpret *Q* as the traceless second-moment tensor of the molecular distribution function. *Q* vanishes in the isotropic phase, whereas this parameter has two degenerate eigenvalues in the uniaxial ordering and can be represented by

(2) $$Q=S\left(\vec{n}⊗\vec{n}-\frac{1}{3}Ι\right)$$

where $\vec{n}$ is the nematic director pointing along the local uniaxial ordering direction, and *S* is the uniaxial scalar parameter expressing the magnitude of fluctuations about the nematic director. In Equation (2), *S* can be either positive or negative. The ensemble of molecules represented by *Q* tends to align along $\vec{n}$ when *S* is positive and tends to lie in the plane orthogonal to $\vec{n}$ when *S* is negative. Finally, the LC is in a biaxial state when all the eigenvalues of *Q* are distinct. The degree of biaxiality is expressed by the parameter β^2^ defined as [20]

(3) $$\beta^{2}=1-\frac{6{\left[tr\left(Q^{3}\right)\right]}^{2}}{{\left[tr\left(Q^{2}\right)\right]}^{3}},$$

This equation is a convenient parameter for illustrating spatial inhomogeneities of *Q* and ranges in the interval [0,1]. All uniaxial states with two degenerate eigenvalues correspond to β^2^ = 0, whereas states with maximal biaxiality correspond to β^2^ = 1. As *tr*(*Q*^3^) = 3det*Q*, states with β^2^ = 1 are those with det *Q* = 0, which implies that at least one eigenvalue of *Q* vanishes.

The Landau–de Gennes free energy density of LC is given by *f* = *f_bulk_*{*Q*_αβ_} + *f_elastic_*{*Q*_αβ_,▽} + *f_dielectric_*{*Q*_αβ_}, in which

(4) $$f_{bulk}=\frac{A}{2}trQ^{2}-\frac{B}{3}trQ^{3}+\frac{C}{4}{\left(trQ^{2}\right)}^{2}$$

is the bulk energy that describes a homogeneous phase. In *f_bulk_*, *B* and *C* are constants, and *A* is assumed to vary with temperature *T* in the form of *A* = *a* (*T* − T*), where *a* is a positive constant and T* is the nematic supercooling temperature. Equation (4) provides the bulk equilibrium value of the uniaxial scalar order parameter in Equation (2) $\;S_{eq}\text{=}\frac{B}{4C}\left(1\text{+}\sqrt{1-\frac{24AC}{B^{2}}}\right)$, which depends on the temperature.

The free-energy *f_elastic_*, which penalizes gradients in the tensor order parameter field, is given in the form

(5) $$f_{elastic}=\frac{1}{2}L{\left|\nabla Q\right|}^{2}$$

where the one-elastic-constant approximation is used for simplicity, and the elasticity of the system is represented by a single positive elastic constant *L.*

The dielectric contribution to the free energy density is given by [21]

(6) $$f_{dielectric}=-\frac{\varepsilon_{0}}{2}\vec{E}\cdot \varepsilon \vec{E}$$

where ε_0_ is the vacuum electric permeability constant, and ε is the dielectric tensor to describe the local anisotropic response of the nematic ordering to $\vec{E}$. ε is generally expressed as

(7) $$\varepsilon =\varepsilon_{i}Ι+\varepsilon_{a}Q$$

where ε*_i_* = (ε*_∥_* + 2ε_⊥_)/3 and ε*_a_ =* (ε*_∥_* − ε_⊥_)/*S_eq_* are isotropic and anisotropic dielectric susceptibilities, respectively. When ε*_a_* > 0, similar to the following calculation, nematic uniaxial ordering is along $\vec{E}$ and therefore *f_dielectric_* can be rewritten as

(8) $$f_{dielectric}=-\frac{\varepsilon_{0}}{2}\left(\varepsilon_{i}{\vec{E}}^{2}+\varepsilon_{a}\vec{E}\cdot Q\vec{E}\right)$$

### 2.2. Geometry of the Problem

**Figure 1:**
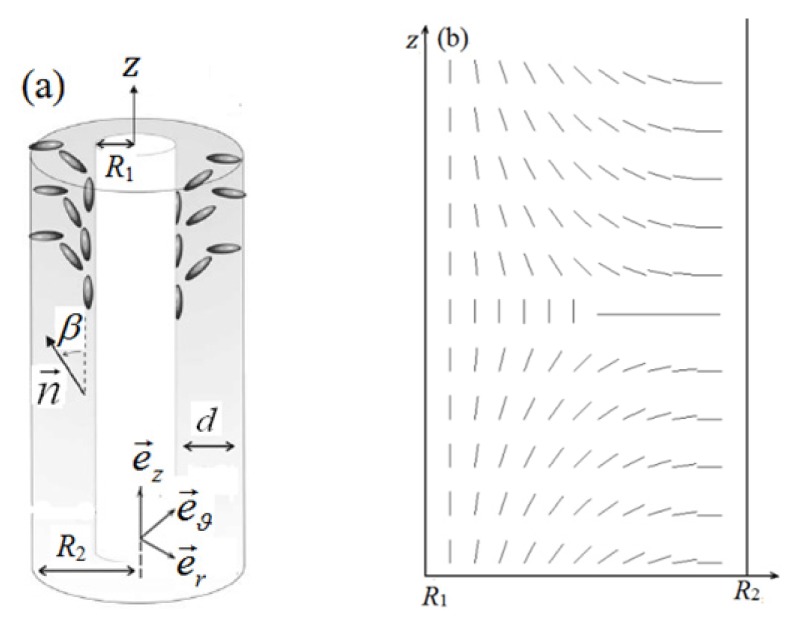
Geometry of the problem. (**a**) Sketch of cylindrical cavity filled with NLCs; and (**b**) the director profile in a cross-section along an arbitrary radius of the system.

Let us consider an NLC confined between two coaxial cylindrical surfaces whose internal and external radii are *R*_1_ and *R*_2_, and the thickness of the shell is *d* = *R*_2_ − *R*_1_ (Figure 1a). The standard polar cylindrical coordinates (r,ϑ,z) and the corresponding local frame $\left({\vec{e}}_{r},{\vec{e}}_{\vartheta },{\vec{e}}_{z}\right)$ are introduced, where ${\vec{e}}_{z}$ points along the symmetry axis, ${\vec{e}}_{r}$ is the radial unit vector emanating from the symmetry axis, and ${\vec{e}}_{\vartheta }:={\vec{e}}_{z}\times {\vec{e}}_{r}$. The electric field $\vec{E}$ is applied along the ${\vec{e}}_{r}$ axis direction, *i.e.*, $\vec{E}=E{\vec{e}}_{r}$, which promotes the alignment of the nematic director along the ${\vec{e}}_{r}$ axis.

LC molecules on the surfaces of internal and external cylinders are strongly anchored along the parallel and perpendicular directions, respectively. According to the investigations of Cavallaro *et al.* [22], we make the assumption that the LC texture exhibits a cylindrical symmetry along the cylindrical axis, *i.e.*, the nematic orientation is independent of ϑ. By varying the polar angle θ_1_ on the internal surface, where θ_1_ is the angle with respect to ${\vec{e}}_{z}$ in the plane ϑ-z, we have verified that the choice of θ_1_ = 0 has the minimum energy, *i.e.*, the director field has no azimuthal component and occurs in the plane *r*-*z*. This is a realistic choice of boundary condition for a cylindrical surface in industrial production. Generally, a homogenous planar alignment is achieved firstly by rubbing on flexible substrate, and then the substrate is fit on the solid cylinder, with the rubbing direction along the symmetric axis. It follows that the texture can be discussed in terms of 2D nematics corresponding to each radial slice. In the plane *r*-*z*, the nematic director is given by $\vec{n}=\left(\sin\beta ,0,\cos\beta \right)$, where â is the angle with respect to ${\vec{e}}_{z}$ (as shown in Figure 1) and the order tensor *Q* can be represented as

(9) $$Q\left(r,z\right)=\left(\begin{array}{ccc} Q_{rr} & 0 & Q_{rz}\\ 0 & Q_{\vartheta \vartheta } & 0\\ Q_{rz} & 0 & Q_{zz} \end{array}\right),$$

Hence, ${\vec{e}}_{\vartheta }$ is always an eigenvector of *Q*. This configuration rules out distortions twisted along ${\vec{e}}_{\vartheta }$. *Q_rz_* in Equation (9) reveals the departures of the eigenframe $\left({\vec{e}}_{1},{\vec{e}}_{2},{\vec{e}}_{3}\right)$ of *Q* from $\left({\vec{e}}_{r},{\vec{e}}_{\vartheta },{\vec{e}}_{z}\right)$, with both frames coinciding when *Q_rz_* = 0.

We denote the free boundary conditions at the upper and lower lateral walls *z* = ±*d_z_*/2, with initial uniaxial ordering by total rotations of −π/2 (*z* = *d_z_*/2) and +*π*/2 (*z* = −*d_z_*/2). These boundary conditions are consistent with the generation of a defect loop with topological charge *s* = −1/2. Figure 1b shows the director profile in a cross-section along an arbitrary radius of the system. The results are also applicable to the defect loop with topological charge *s* = 1/2, with exchanged conditions of the upper and lower walls.

### 2.3. Scaling and Dimensionless Evolution Equations

We introduced the following dimensionless quantities: $\tilde{f}\equiv f/\left[\frac{B^{4}}{{\left(4C\right)}^{3}}\right]$, ${\tilde{Q}}_{ij}\equiv Q_{ij}/q_{0}$, $\tilde{z}\equiv {z/\xi }_{0}$, $\tilde{r}\equiv {r/\xi }_{0}$, ${\tilde{\varepsilon }}_{a}=\varepsilon_{a}q_{0}/\varepsilon_{i}$, $E_{0}=\left(q_{0}/\xi_{0}\right)\sqrt{2L/\tilde{A}\varepsilon_{i}\varepsilon_{0}}$, $\tilde{E}=E/E_{0}$, where $q_{0}=B/4C$ is the superheating order parameter at the nematic superheating temperature T**, and $\xi_{0}=\sqrt{\frac{L}{Bq_{0}}}=\sqrt{\frac{4CL}{B^{2}}}$ is the characteristic length for order-parameter changes. Equations (4), (5), and (8) can be reduced to

(10) $${\tilde{f}}_{bulk}=\frac{\tilde{A}}{12}tr{\tilde{Q}}^{2}-\frac{1}{3}tr{\tilde{Q}}^{3}+\frac{1}{16}{\left(tr{\tilde{Q}}^{2}\right)}^{2}$$

(11) $${\tilde{f}}_{elastic}=\frac{1}{2}{\left|\tilde{\nabla }\tilde{Q}\right|}^{2}$$

(12) $${\tilde{f}}_{dielectric}=-\frac{1}{\tilde{A}}\left[{\tilde{E}}^{2}+{\tilde{\varepsilon }}_{a}{\tilde{Q}}_{rr}{\tilde{E}}^{2}\right]$$

where the reduced parameter $\tilde{A}=\frac{24AC}{B^{2}}$ defines the temperature scale. Isotropic–nematic transition occurs at $\tilde{A}=8/9$. In polar cylindrical coordinates, Equation (11) can be expressed precisely by

(13) $$\begin{array}{cc} {\tilde{f}}_{e}= & \frac{1}{2}\left[{\left(\frac{\partial {\tilde{Q}}_{rr}}{\partial \tilde{r}}\right)}^{2}+{\left(\frac{\partial {\tilde{Q}}_{rr}}{\partial \tilde{z}}\right)}^{2}+{\left(\frac{\partial {\tilde{Q}}_{\vartheta \vartheta }}{\partial \tilde{r}}\right)}^{2}+{\left(\frac{\partial {\tilde{Q}}_{\vartheta \vartheta }}{\partial \tilde{z}}\right)}^{2}+{\left(\frac{\partial {\tilde{Q}}_{zz}}{\partial \tilde{r}}\right)}^{2}+{\left(\frac{\partial {\tilde{Q}}_{zz}}{\partial \tilde{z}}\right)}^{2}\right]\\ \; & +\left[{\left(\frac{\partial {\tilde{Q}}_{rz}}{\partial \tilde{r}}\right)}^{2}+{\left(\frac{\partial {\tilde{Q}}_{rz}}{\partial \tilde{z}}\right)}^{2}\right]+\frac{1}{{\tilde{r}}^{2}}\left({{\tilde{Q}}_{rr}}^{2}+{{\tilde{Q}}_{\vartheta \vartheta }}^{2}+{{\tilde{Q}}_{rz}}^{2}-2{\tilde{Q}}_{rr}{\tilde{Q}}_{\vartheta \vartheta }\right)\;. \end{array}$$

Thus, the reduced uniaxial ordering has the form

(14) $$\tilde{Q}=\tilde{S}{\left(3\vec{n}⊗\vec{n}-Ι\right)}^{2},$$

where $\tilde{s}=\frac{S_{eq}}{q_{0}}=1+\sqrt{1-\tilde{A}}$ is the reduced uniaxial scalar parameter at equilibrium.

We compute the evolution of LC with dynamic theory for tensor order-parameter field *Q*(*r*,z,*t*). The local values of the scalar-order parameter *S* and the director $\vec{n}$ can be calculated from *Q* by using the highest eigenvalue and the associated eigenvector, respectively. According to [23], the evolution equation describing the dynamics of *Q* can be written as

(15) $$\frac{\partial Q}{\partial t}=\Gamma \left[-\frac{\delta f}{\delta Q}+\frac{1}{3}tr\left(\frac{\delta f}{\delta Q}\right)Ι\right]\text{,}$$

where Γ = 6D*/[1 − 3*tr*(*Q*^2^)]^2^, D* is the rotational diffusion for the nematic, and δf/δQ is assumed to be symmetrical.

Numerical calculations are performed using the reduced variables. When the functional derivatives in Equation (15) are evaluated, the following evolution equations for $\tilde{Q}$ can be obtained.

(16) $$\begin{array}{l} \frac{\partial {\tilde{Q}}_{rr}}{\partial t}=\tilde{\Gamma }[-\frac{\tilde{A}}{6}{\tilde{Q}}_{rr}+{\left({\tilde{Q}}^{2}\right)}_{rr}-\left(tr{\tilde{Q}}^{2}\right)\left(\frac{{\tilde{Q}}_{rr}}{4}+\frac{1}{3}\right)-\frac{2}{{\tilde{r}}^{2}}\left({\tilde{Q}}_{rr}-{\tilde{Q}}_{\vartheta \vartheta }\right)\\ \;+{\tilde{Q}}_{rr,\tilde{r}\tilde{r}}+\frac{1}{\tilde{r}}{\tilde{Q}}_{rr,\tilde{r}}+{\tilde{Q}}_{rr,\tilde{z}\tilde{z}}], \end{array}$$

(17) $$\begin{array}{l} \frac{\partial {\tilde{Q}}_{\vartheta \vartheta }}{\partial t}=\tilde{\Gamma }[-\frac{\tilde{A}}{6}{\tilde{Q}}_{\vartheta \vartheta }+{\left({\tilde{Q}}^{2}\right)}_{\vartheta \vartheta }-\left(tr{\tilde{Q}}^{2}\right)\left(\frac{{\tilde{Q}}_{\vartheta \vartheta }}{4}+\frac{1}{3}\right)-\frac{2}{{\tilde{r}}^{2}}\left({\tilde{Q}}_{\vartheta \vartheta }-{\tilde{Q}}_{rr}\right)\\ \;+{\tilde{Q}}_{\vartheta \vartheta ,\tilde{r}\tilde{r}}+\frac{1}{\tilde{\rho }}{\tilde{Q}}_{\vartheta \vartheta ,\tilde{r}}+{\tilde{Q}}_{\vartheta \vartheta ,\tilde{z}\tilde{z}}], \end{array}$$

(18) $$\frac{\partial {\tilde{Q}}_{zz}}{\partial t}=\tilde{\Gamma }\left[-\frac{\tilde{A}}{6}{\tilde{Q}}_{zz}+{\left({\tilde{Q}}^{2}\right)}_{zz}-\left(tr{\tilde{Q}}^{2}\right)\left(\frac{{\tilde{Q}}_{zz}}{4}+\frac{1}{3}\right)+{\tilde{Q}}_{zz,\tilde{r}\tilde{r}}+\frac{1}{\tilde{r}}{\tilde{Q}}_{zz,\tilde{r}}+{\tilde{Q}}_{zz,\tilde{z}\tilde{z}}\right],$$

(19) $$\frac{\partial {\tilde{Q}}_{rz}}{\partial t}=\tilde{\Gamma }\left[-\frac{\tilde{A}}{3}{\tilde{Q}}_{rz}+2{\left({\tilde{Q}}^{2}\right)}_{rz}-\frac{1}{2}\left(tr{\tilde{Q}}^{2}\right){\tilde{Q}}_{rz}-\frac{2{\tilde{Q}}_{rz}}{{\tilde{r}}^{2}}+2{\tilde{Q}}_{rz,\tilde{r}\tilde{r}}+\frac{1}{\tilde{r}}2{\tilde{Q}}_{rz,\tilde{r}}+2{\tilde{Q}}_{rz,\tilde{z}\tilde{z}}\right].$$

with $\tilde{\Gamma }=\Gamma \times \left(Bq_{0}\right)$. We adopt the two-dimensional finite-difference method developed in our previous studies [24,25] to obtain the numerical simulation results. Here, we let the system relax from the initial boundary conditions given in Section 2.2, and the initial conditions of the upper and lower half in the bulk are specified by total rotations of −π/2 and +*π*/2, respectively, consistent with the boundary conditions at the upper and lower walls given above. We consider only the equilibrium configurations that correspond to the global minimum *F*. In our numerical calculations, a discretization with a time step given by 10^−10^ is sufficient to guarantee the stability of the numerical procedure. In addition, our equilibration runs are 2 × 10^6^, which is adequate for the system to reach the equilibrium.

## 3. Results

According to parameters given in [26], *a* = 0.195 × 10^6^ J/m^3^, *B* = 7.155 × 10^6^ J/m^3^, *C* = 8.82 × 10^6^ J/m^3^, *L* = 10.125 × 10^−12^ J/m can be easily obtained. In our simulations, the scaled temperature is set to $\tilde{A}=2/3$, which corresponds to $\tilde{s}=1+\sqrt{1/3}$. The rotational diffusion D* is set to 0.35, which is the value used in [26], and the dielectric anisotropy is set to Δε = 13.2 (ε_⊥_ = 6.5). We then obtain the exact values ξ_0_ = 2.64 *nm* and *E*_0_ = 43.1 *V*/*um*. We focus on the dependence of structure transition on thickness (*d*) and then on the structure transition induced by electric field ($\vec{E}$).

### 3.1. Structure Transition with Different Values of Thickness d and Internal Radius R_1_

**Figure 2:**
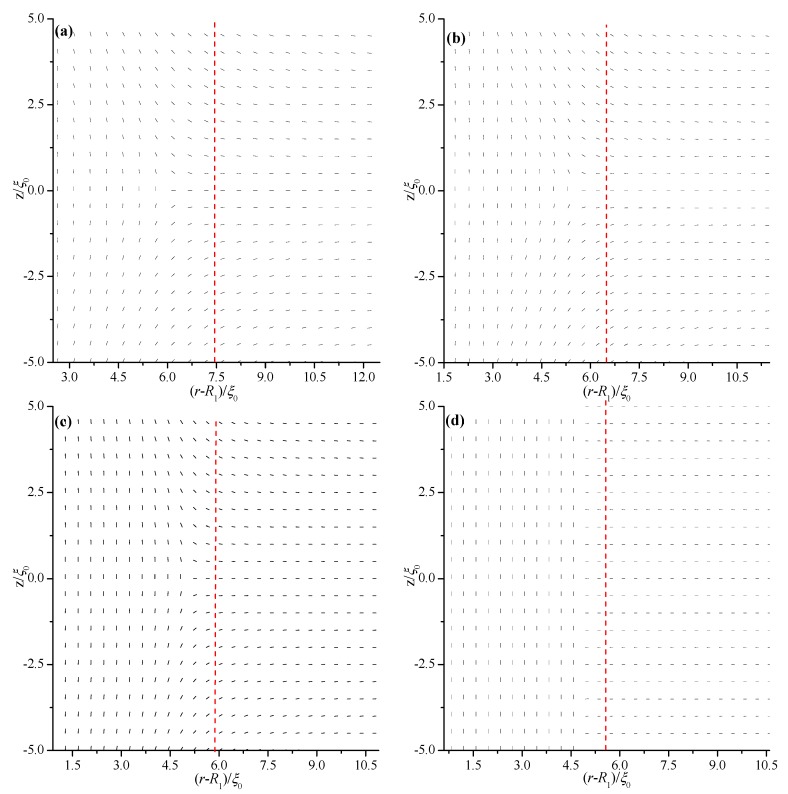
Director field profile at the equilibrium state in a cross-section along an arbitrary azimuth inside the coaxial cylindrical system for *R*_1_ = 30 *ξ*_0_ with different thicknesses (*d*). The red dotted lines represent the middle of the simulation system. (**a**) *d* = 15 *ξ*_0_; (**b**) *d* = 13 *ξ*_0_; (**c**) *d* = 11.8 *ξ*_0_; and (**d**) *d* = 11.2 *ξ*_0_.

**Figure 3:**
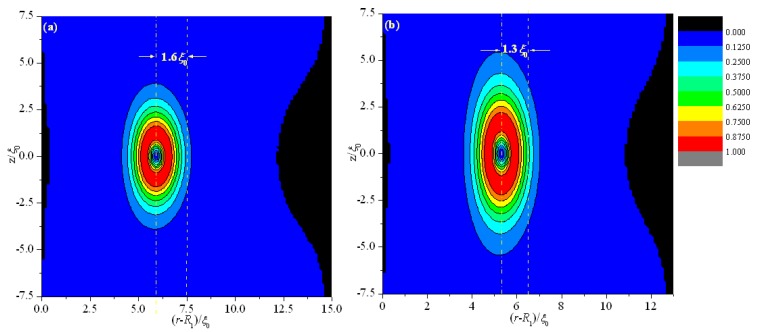

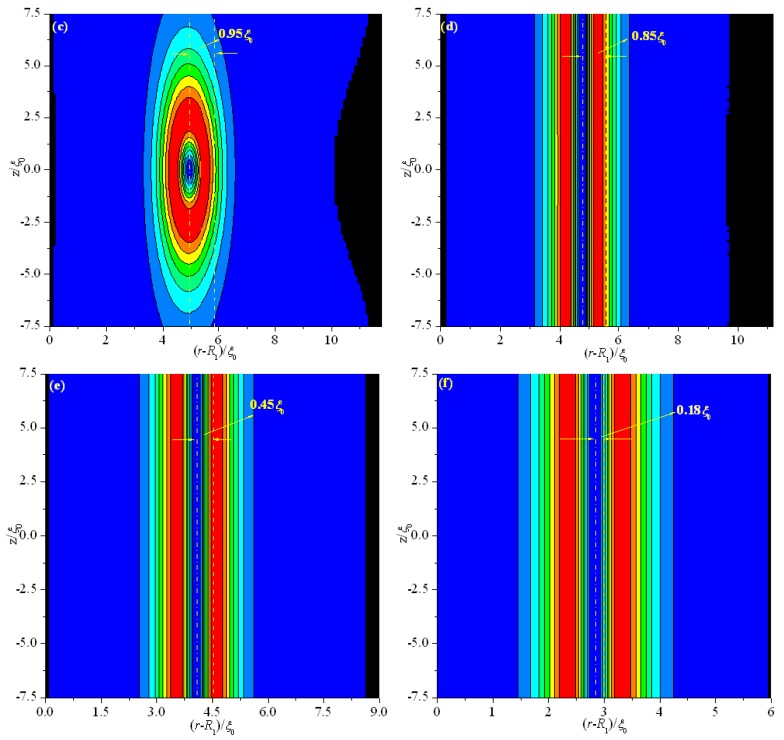
Biaxiality β^2^ for different thicknesses (*d*) in a cross-section along an arbitrary azimuth inside the coaxial cylindrical system for *R*_1_ = 30 ξ_0_. The dotted and dashed lines represent the middle of the simulation system and the center of the defect, respectively. (**a**) *d* = 15 ξ_0_; (**b**) *d* = 13 ξ_0_; (**c**) *d* = 11.8 ξ_0_; (**d**) *d* = 11.2 ξ_0_; (**e**) *d* = 9 ξ_0_; and (**f**) *d* = 6 ξ_0_.

We first analyze the equilibrium textures in the case of *R*_1_ = 30 ξ_0_ with different values of *d*. Figure 2 and Figure 3 show the director field profile and the calculated biaxiality β^2^ in a cross-section along an arbitrary azimuth for different values of *d*. At relatively large thicknesses, the nematic system shows the defect core structure (Figure 2a,b) and is uniaxial everywhere (β^2^ = 0), except for a small region around the defect core (Figure 3a,b). When *d* decreases, the defect core clearly explodes along *z* (Figure 2c), and a large biaxiality propagates along *z* inside the system (Figure 3c). Further decrease in *d* results in the creation of a biaxial wall, which connects the two orthogonal uniaxial directions imposed by two cylindrical surfaces (Figure 2d and Figure 3d) at a critical value of *d*_c_ = 11.2 ξ_0_. In summary, the system has transitioned from the eigenvector rotation configuration with a defect loop into the eigenvalue exchange/order reconstruction configuration by developing a thin biaxial nematic layer, which forms a cylindrical wall in the nematic system. The transition process is similar to that conducted in a planar cell in our previous studies [24,25].

Figure 2 and Figure 3 show that the defect center is not located in the middle of the system but shifts to the internal surface; this phenomenon differs from the case of a planar cell [24,25]. Non-planar cylindrical geometry causes disclination loops to shrink, and the loop will not disappear under internal surface confinement [27]. Figure 3 shows that the deviations Δ(*d*) for different thicknesses (*d*) are 1.6 ξ_0_ (*d* = 15 ξ_0_), 1.3 ξ_0_ (*d* = 13 ξ_0_), 0.95 ξ_0_ (*d* = 11.8 ξ_0_), 0.85 ξ_0_ (*d* = 11.2 ξ_0_), 0.45 ξ_0_ (*d* = 9 ξ_0_), and 0.18 ξ_0_ (*d* = 6 ξ_0_). These finding indicate that Δ(*d*) decreases with decreasing *d*, even after eigenvalue exchange (Figure 3e,f). This behavior is due to the gradual transformation of the system to a planar structure with decreasing *d*.

**Figure 4:**
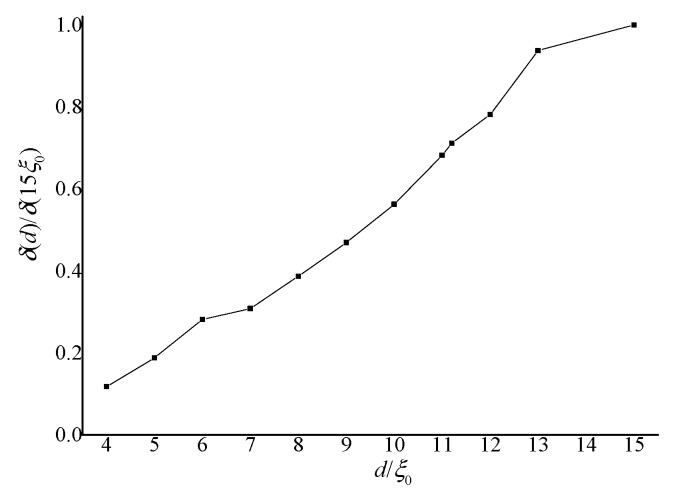
Curves of calculated δ(*d*)/δ(15 ξ_0_) as a function of *d*/ξ_0_.

The relative deviations δ(d) = Δ(d)/d are calculated and normalized by δ(d)/δ(15 ξ_0_) to analyze the influence of non-planar geometry. Figure 4 shows the curves of δ(d)/δ(15 ξ_0_) as a function of d/ ξ_0_. In the figure, the relative deviations decrease with decreasing thickness *d*.

A clearer explanation of this phenomenon is as follows: For a planar hybrid-aligned cell (*R*_1_→∞), the defect center is located in the middle of the system, with the energy on both sides of the defect equal. While for the non-planar cylindrical geometry, if the director configuration keeps the configuration of a planar cell, the energy on the inner side of the defect will be lower than that on the outer side, because that the volume on the inner side is smaller, relative to the outer side. Thus the defect center shifts to the inner side, and the director configuration of the nematic meanwhile changes, until the system reaches equilibrium. For fixed *R*_1_, the volume differences on the two sides of the middle of the system decrease with decreasing *d*, thus deviations decrease with decreasing *d*.

The energy calculation shows that the energy in the “inner” region of the cylindrical geometry is higher than that in the “outer” region, indicating that more energy can be saved by shrinking the disclination to a smaller radius to reduce its total length. In addition, the shrinking of the disclination to a smaller radius also helps to decrease the total energy of the system.

**Figure 5:**
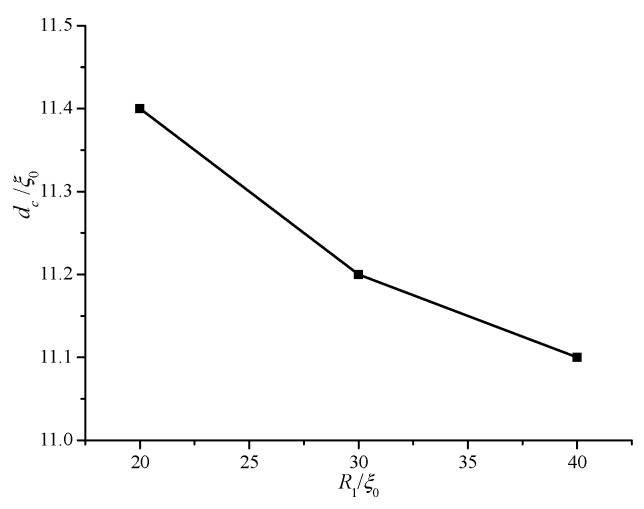
Critical values of *d_c_* for different internal radii (*R*_1_).

The conditions of *R*_1_ = 20 ξ_0_ and *R*_1_ = 40 ξ_0_ are also investigated to explore the influence of internal radius *R*_1_ on equilibrium texture and transition. The critical *d_c_* values for different *R*_1_ values are shown in Figure 5. The value of *d_c_* increases with decreasing *R*_1_. This behavior indicates that the smaller the internal radius *R*_1_, the earlier the biaxial transition occurs. Therefore, a non-planar cylindrical system induces easy biaxial transition. The cylindrical system gradually flattens with increasing *R*_1_. Thus, we predict that *d*_c_ gradually becomes equal to that of a planar cell with increasing *R*_1_.

**Figure 6:**
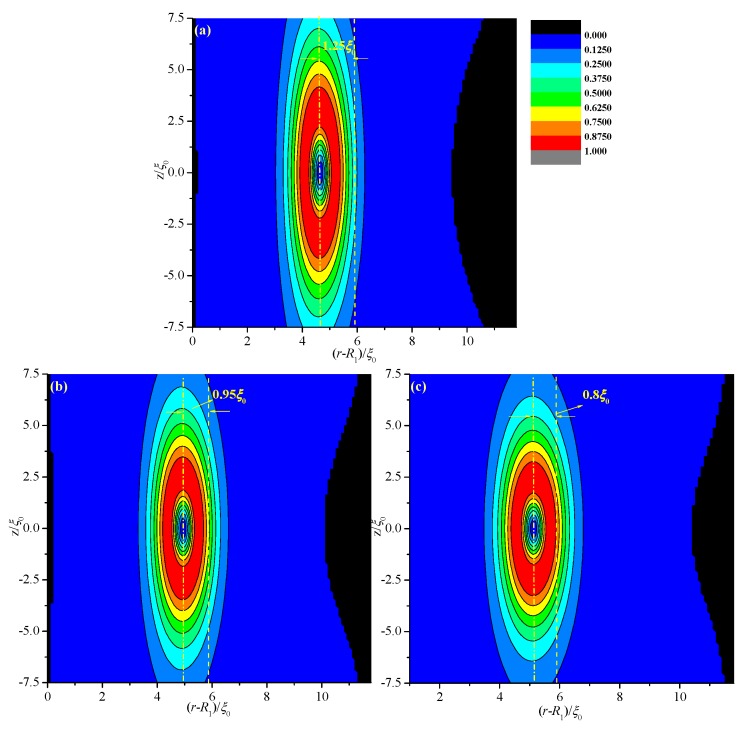
Biaxiality β^2^ in a cross-section along an arbitrary azimuth inside the coaxial cylindrical system for different internal radii (*R*_1_) with a certain thickness *d* = 11.8 *ξ*_0_. The dotted and dashed lines represent the middle of the simulation system and the center of the defect, respectively. (**a**) *R*_1_ = 20 ξ_0_; (**b**) *R*_1_ = 30 ξ_0_; and (**c**) *R*_1_ = 40 ξ_0_.

Figure 6 shows the biaxiality β^2^ in a cross-section for different values of *R*_1_ with fixed thickness *d* = 11.8 ξ_0_. Deviations Δ(*d*) are 1.25 ξ_0_ (*R*_1_ = 20 ξ_0_), 0.95 ξ_0_ (*R*_1_ = 30 ξ_0_), and 0.8 ξ_0_ (*R*_1_ = 40 ξ_0_); this finding indicates that Δ(*d*) decreases with increasing *R*_1_ or with gradual flattening of the system. That is because for fixed *d*, the volume ratio on the two sides of the middle of the system decreases with increasing *R*_1_. We predict that Δ(*d*) gradually approaches zero when *R*_1_ approaches infinity.

### 3.2. Structure Transition Induced by Electric Field $\vec{E}$

**Figure 7:**
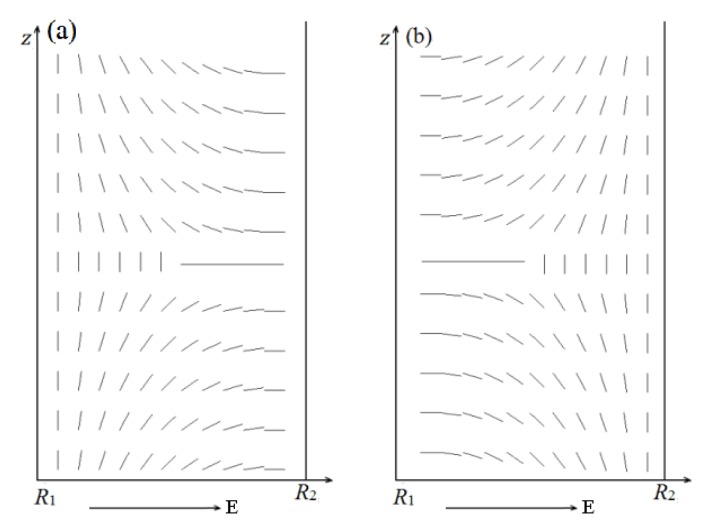
Director profile in a cross-section along an arbitrary radius of the system for the two models. (**a**) Models I and (**b**) II.

In this section, two models are established to analyze the combined effect of geometry and electric field $\vec{E}$. Boundaries on both coaxial cylindrical surfaces are exchanged for the two models, as shown in Figure 7. Simulations are conducted at internal radius *R*_1_ = 30 *ξ*_0_ and thickness *d* = 15 *ξ*_0_.

**Figure 8:**
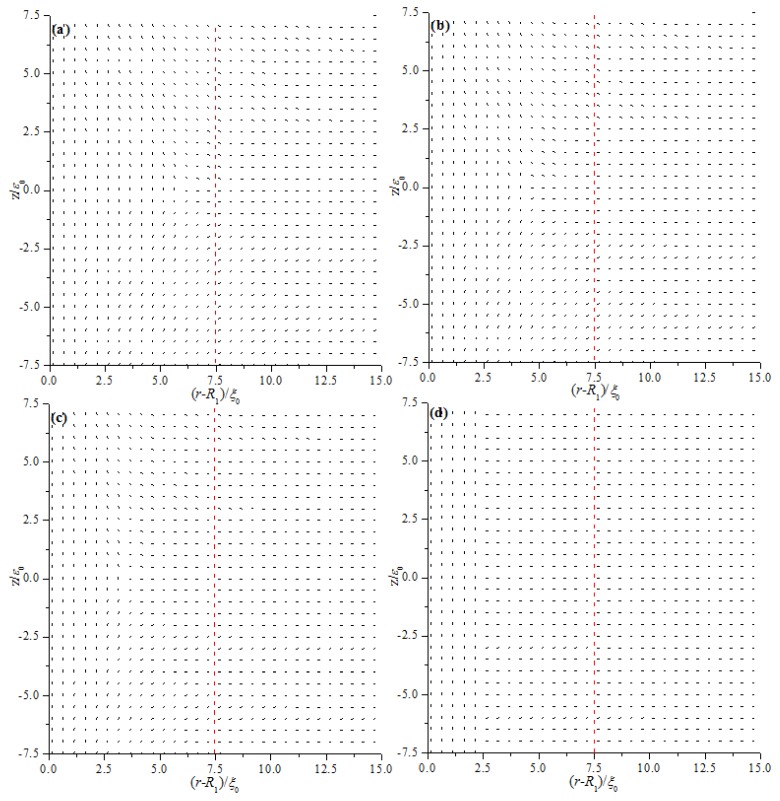
Director field profile at the equilibrium state in a cross-section along an arbitrary azimuth inside the cylindrical system for different $\tilde{E}$ values for model I. (**a**) $\tilde{E}=0$; (**b**) $\tilde{E}=0.1$; (**c**) $\tilde{E}=0.15$; and (**d**) $\tilde{E}=0.21$.

**Figure 9:**
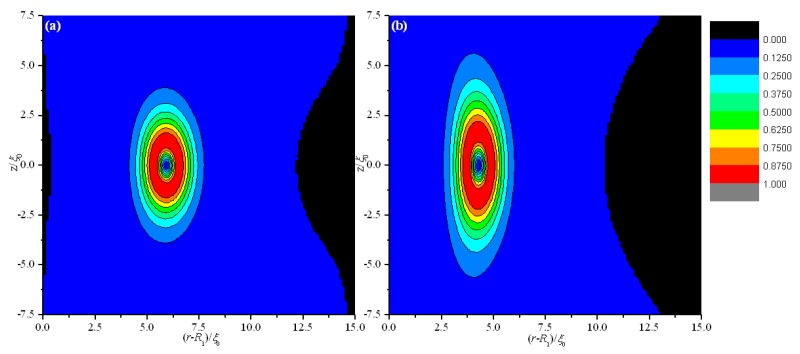

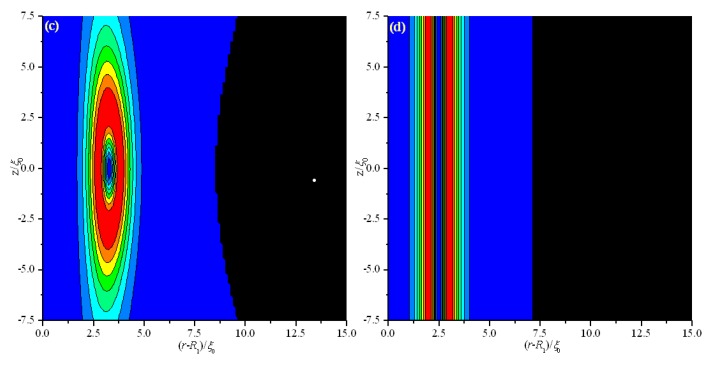
Biaxiality β^2^ in a cross-section along an arbitrary azimuth inside the cylindrical system for different $\tilde{E}$ values for model I. (**a**) $\tilde{E}=0$; (**b**) $\tilde{E}=0.1$; (**c**) $\tilde{E}=0.15$; and (**d**) $\tilde{E}=0.21$.

**Figure 10:**
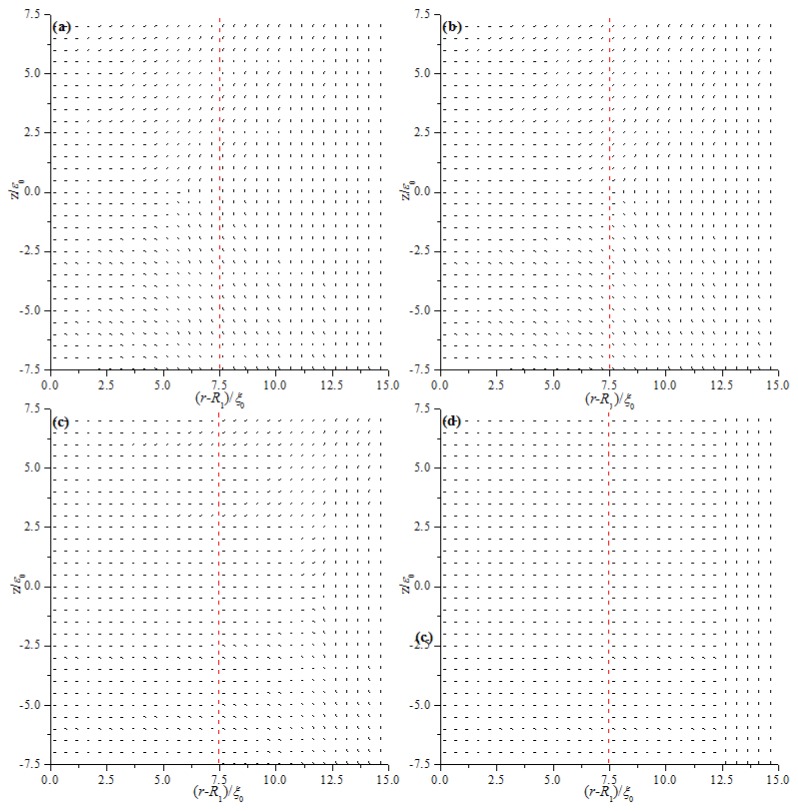
Director field profile at the equilibrium state in a cross-section along an arbitrary azimuth inside the cylindrical system for different $\tilde{E}$ values model II. (**a**) $\tilde{E}=0$; (**b**) $\tilde{E}=0.1$; (**c**) $\tilde{E}=0.2$; and (**d**) $\tilde{E}=0.25$.

**Figure 11:**
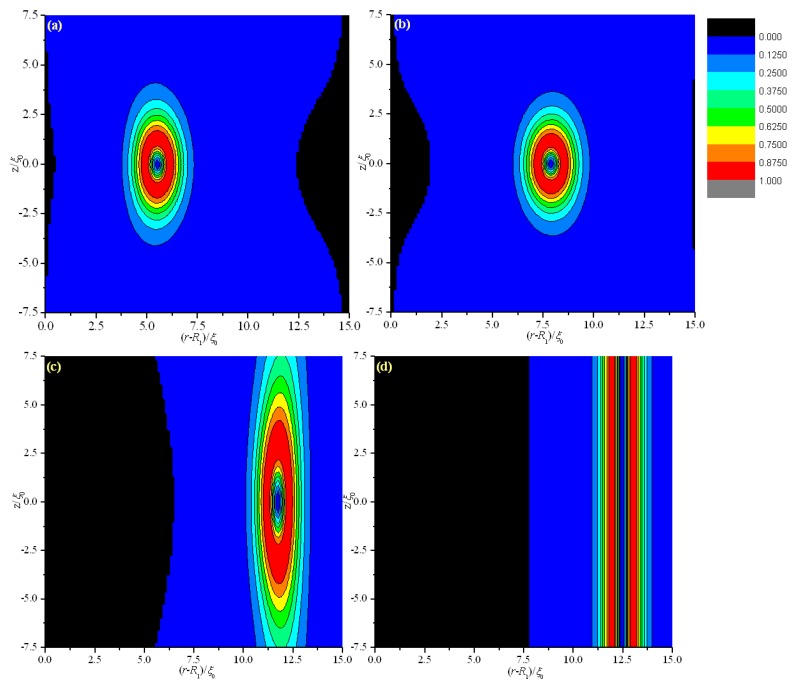
Biaxiality β^2^ in a cross-section along an arbitrary azimuth inside the cylindrical system for different $\tilde{E}$ values for model II. (**a**) $\tilde{E}=0$; (**b**) $\tilde{E}=0.1$; (**c**) $\tilde{E}=0.2$; and (**d**) $\tilde{E}=0.25$.

Figure 8 and Figure 9 show the director field profile and the calculated biaxiality β^2^ in a cross-section along an arbitrary azimuth as a function of $\tilde{E}$ for model I, and Figure 10 and Figure 11 show the results for model II, respectively. The disclination loops shift to the internal surface at $\tilde{E}=0$, which is caused by the non-planar cylindrical geometry. The defect is pushed further toward the internal surface for model I with increasing $\tilde{E}$, whereas the defect initially shifts to the middle of the system and then to the external surface for model II. Because of the strong anchoring boundaries, the defects exhibit dramatic changes in shape as the distance between defect center and the surface boundary decreasing, especially when the defect center lies very close to the surface, a biaxial layer is established, that is order reconstruction occurs [9,24,25]. Our results show that order reconstruction occurs at critical values of ${\tilde{E}}_{c}\left(Ι\right)=$ 0.21 and ${\tilde{E}}_{c}\left(ΙΙ\right)=$ 0.25 for models I and II, respectively. For comparison, a planar cell is also simulated with the same parameters, and the corresponding reduced critical value of electric field is ${\tilde{E}}_{c0}=$ 0.24. The values are clearly ranked as follows: ${\tilde{E}}_{c}\left(Ι\right)<{\tilde{E}}_{c0}<{\tilde{E}}_{c}\left(ΙΙ\right)$.

Non-planar cylindrical geometry induces disclination loops to shrink for both models, whereas electric field $\vec{E}$, which tends to enforce the nematic director along the ${\vec{e}}_{r}$ axis, expulses the defect to the internal and external surfaces for models I and II, respectively. It means that the effects of the cylindrical geometry and electric field $\vec{E}$ are common for model I, but opposite for model II. Hence, the transition process differs between the two models. The common action of the cylindrical geometry and electric field $\vec{E}$ for model I makes the defect close to the surface boundary more easily than model II, since the opposite action of the cylindrical geometry and electric field $\vec{E}$ makes it difficult for the defect to be close to the surface boundary. From the above, the common action of the cylindrical geometry and electric field $\vec{E}$ for model I facilitates order reconstruction, while the opposite action of the cylindrical geometry and electric field $\vec{E}$ for model II complicates order reconstruction. For a planar cell, only the action of electric field $\vec{E}$ exists. Considering these factors, our result ${\tilde{E}}_{c}\left(Ι\right)<{\tilde{E}}_{c0}<{\tilde{E}}_{c}\left(ΙΙ\right)$ is reasonable.

Note that the radial direction uniform electric field has been assumed, which is difficult to implement or almost impossible in practice. Our results only give the qualitative analysis about the effect of the electric field as well as the combined effect of geometry and the electric field. We can conclude that for the actual cylindrical symmetric radial direction electric field $E\left(r\right)=\frac{E_{0}}{r}$, the same conclusion is reached in nature. The detailed and definite results for the actual cylindrical symmetric radial direction electric field are a task for the future.

## 4. Conclusions

Structural transitions of an NLC confined between two coaxial cylinders were investigated through Landau–de Gennes theory by using a two-dimensional finite-difference iterative method. We considered the cylindrical symmetry configuration about the cylindrical axis. The effects of the shell thickness *d*, as well as the strength of the electric field $\vec{E}$, were also analyzed. The results show that order reconstruction occurs in a thin enough shell or under a strong enough electric field. The non-planar system causes the defect center to deviate from the middle of the system to the internal surface. The common action of the electric field $\vec{E}$ along the ${\vec{e}}_{r}$ axis and the non-planar geometry facilitates order reconstruction, whereas their opposite action complicates the process.

By using the obtained parameters, we easily determined the characteristic length *ξ*_0_ ~ 2.64 nm and the critical separation *d*_c_ ~ 30 nm, which is higher than our simulation result for a planar cell (~27 nm) [24,25]. The non-planar cylindrical system facilitates biaxial transition. We speculate that *d*_c_ gradually becomes equal to that of a planar cell with increasing *R*_1_.

One thing should be pointed out specially here. In our investigation of the effect of thickness d and internal radius *R*_1_ in Section 3.1, only the effect of non-planar geometry exists, and the results for the two models are similar. Thus we give the results of Model I only in our manuscript.

Various structures of LC cells may be designed to improve the optical characteristics of an LC cell. Different directions of $\vec{E}$ may also be applied and may yield unpredictable optical abnormalities because of the generated defects. The evident effect of $\vec{E}$ on the dynamic behavior of LC around the defects and transitions between the topologically different states can contribute to the design of advanced LC modes.

Our simulation describes an isolated and stabilized defect reasonably well. A natural question is whether our simulation describes a realistic scenario within such a coaxial cylindrical nematic system. In fact, defect rings with alternating winding signs exist when the defect spacing in the z direction is large enough. With defect spacing decreasing, the defect rings gradually annihilate at a characteristic length. Identifying the detailed effects of the defect spacing in the z direction as well as the electric field on the defect structure are tasks for the future.

Comparison of experimental works with numerical results in nematic shells is rare because shells are difficult to produce in a controlled manner. Thus far, microfluidics has provided a natural method to overcome this limitation. Nevertheless, future theoretical and experimental investigations must be performed to determine the effects of electromagnetic field, temperature, or boundary anchoring on nematic shells and even smectic or cholesteric shells, especially when degenerate planar anchoring condition is prescribed on the cylinder surface; this would be a good and interesting topic. In addition, the geometry of a bookshelf on curved surfaces is also an interesting task for the future.
